# The evolution of transcarotid artery stenting with flow reversal

**DOI:** 10.1590/1677-5449.190105

**Published:** 2019-10-23

**Authors:** Rafael Demarchi Malgor, Rasesh Mahendra Shah

**Affiliations:** 1 University of Colorado, Division of Vascular Surgery, Aurora, USA.; 2 Eastern Virginia Medical School, Division of Vascular Surgery, Norfolk, USA.

Stroke remains an important economic and social burden in western societies. The most common cause of stroke is an embolic event, which can originate from several different sources (i.e. carotid atheroma, arch atheroma, valvular heart disease, or atrial fibrillation). The annual risk of developing any cerebrovascular event in patients with a carotid atheroma is 1-2%, based on the Asymptomatic Carotid Atherosclerosis Study (ACAS) and the Asymptomatic Carotid Surgery Trial (ACST).^1^

Carotid revascularization can be performed by open, endovascular, or hybrid approaches. For decades, carotid endarterectomy (CEA), first performed by Dr. Michael DeBakey in 1953, was the only carotid revascularization procedure proven to prevent stroke. Almost 25 years later, the advent and refinements of endovascular therapy made it possible for the first percutaneous carotid artery angioplasty to be performed in 1977 by Dr. Mathias. However, the risk of distal embolization, and therefore stroke, during unprotected carotid angioplasty remained a problem until the development of neuro protection systems in the late 1990s. Knowing about the evolution of cerebral protection during carotid artery revascularization is paramount to understanding the current commercially available devices. Juan Parodi carried out the first study on neuroprotection during carotid artery endovascular revascularization in 1998.^2^ His initial studies focused on determining embolization rate to the brain during CEA using transcranial Doppler (TCD). TCD had been previously utilized to monitor microembolic signals (MES) during CEA in the middle cerebral artery.^3^

The same concept of monitoring MES during CEA was then extrapolated to percutaneous carotid artery procedures. Occlusion of the distal internal carotid artery was the first method employed to prevent distal embolization during carotid artery angioplasty.^4^ Devices providing concomitant balloon occlusion of the common carotid artery (CCA) and the ECA were fashioned by Dr. Parodi and Dr. Coppi, decreasing the MES detected by TCD during carotid artery angioplasty.^5^^,^ 6 A few years later, Dr Yadav introduced the concept of an ICA filter device (Angioguard™, Cordis, Milpitas, CA).^7^ Unfortunately, microparticle embolization to the brain was nevertheless detected using TCD, as the filter device has to cross the carotid artery lesion before it is deployed. A flow reversal system which can continuously aspirate these microparticles, filter and return the blood through a venous access was thought to be the answer to preventing brain embolization.

The first flow reversal system was designed by Dr. Parodi and tested by Dr. Bates. Initially, continuous passive aspiration through a side port was created and flow reversal was confirmed by a contralateral carotid artery angiography, which confirmed retrograde flow in the ipsilateral carotid artery. Due to difficulties with complex aortic arch configurations, arch atheroma, and the need for ECA balloon placement in addition to CCA access to achieve true flow reversal, the transfemoral approach was abandoned and focus was once more directed to the transcervical approach. A combination of flow reversal and filter protection was also utilized in the early 2000s,^8^ but with the improvements in flow reversal devices filters are no longer needed.

Feasibility studies were then conducted in which flow reversal was achieved by connecting commercially available 8Fr sheaths placed in the carotid artery and the jugular vein.^9^ In 2004, the first series of patients presenting with symptomatic and asymptomatic carotid artery stenosis treated with TCAR with flow reversal via connecting sheaths placed in the carotid artery and jugular vein was published by Criado et al.^10^ Seven of the 10 patients included were symptomatic and all patients were deemed to be high-risk for CEA. One patient had transient upper extremity weakness, which was related to a contralateral ICA occlusion during flow reversal. The patient eventually recovered. In the same year, Chang et al. reported their experience of 21 patients undergoing TCAR.^11^ A small modification to the technique utilized by Parodi and Bates and later by Criado was use of a 60-μm filter connected to the 9Fr CCA sheath to retain debris prior to returning the blood into the jugular vein through a 6Fr sheath. Again, no 30-day major strokes or death were reported; no access site complications were found.

After Criado’s report of his technique and results, Alexandrescu et al.^12^ modified the flow reversal approach during TCAR. Twenty-six patients underwent CAS through a transcervical approach; all patients had a filter placed into the ICA while flow reversal was carried out. Flow reversal was utilized only during filter placement. Stroke/death was reported to be zero and only 1 minor ipsilateral stroke occurred. In 2007, Criado et al.^13^ published their 3-year follow-up experience, which included 103 stents. In that large series, no major strokes or death were noted; one ipsilateral and one contralateral transient ischemic attack (TIA) along with two minor strokes were reported. Three (4%) patients did not tolerate flow reversal.

To date, two pivotal multicenter trials designed to investigate CAS under neuroprotection with flow reversal have been published.^14^^,^ 15 In the first trial, Clair et al.^15^ reported the EMPiRE Clinical Study 30-day outcomes; twenty-nine centers enrolled 245 patients to investigate the efficacy and safety of the GORE Flow Reversal System™ (W.L. Gore, Flagstaff, AZ), which is based on Parodi’s first flow reversal system (ECA and CCA balloon occlusion through a femoral artery approach and blood return to the femoral vein). Despite acceptable outcomes, the device was later discontinued by the company based on market analysis.

**Figure 1:**
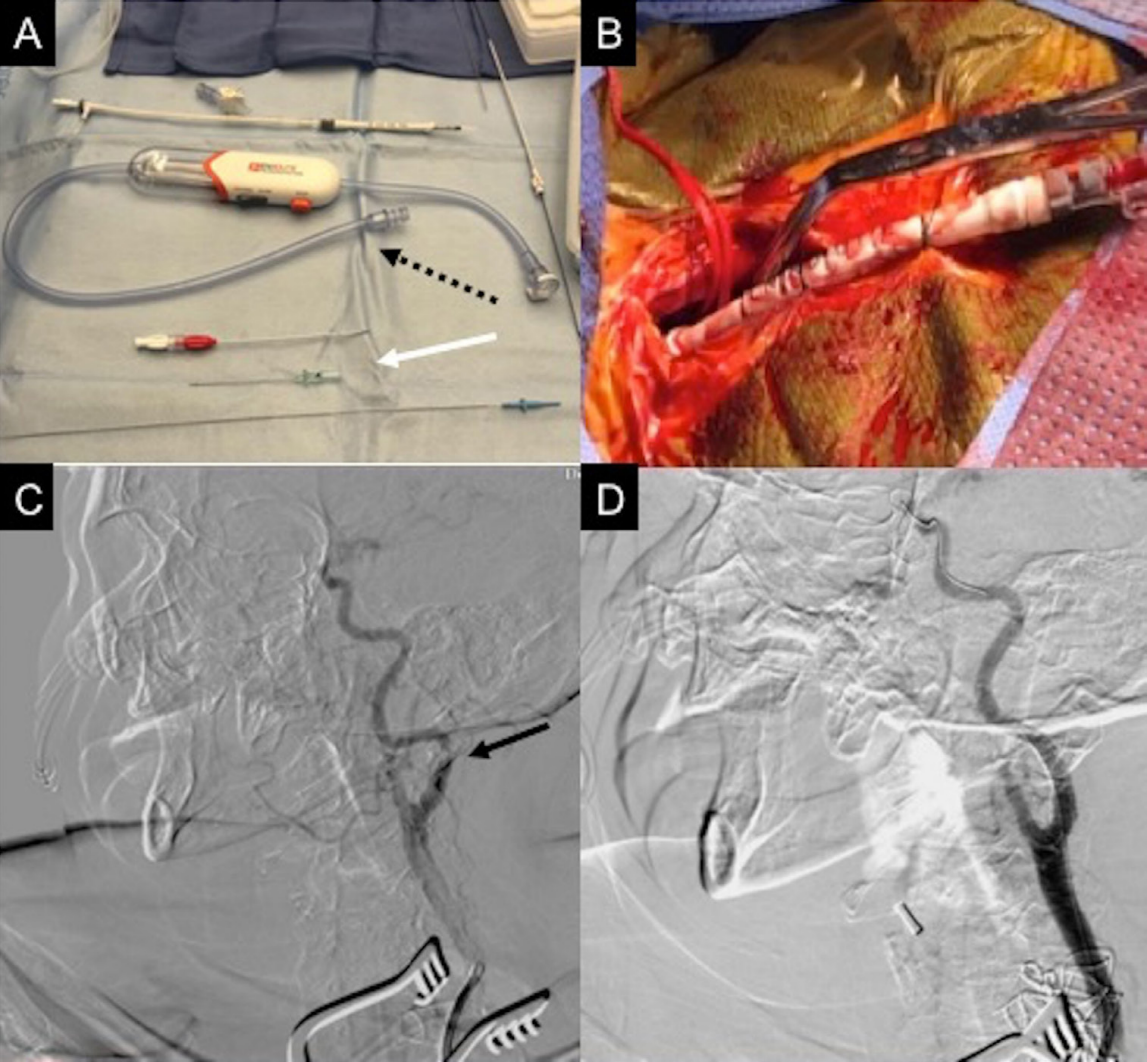
76-year-old female presenting with transient ischemic attack (right arm weakness). (A) Enroute™ neuroprotection system (Silk Road, California, US) with flow reversal/filter device (white arrow) and micro puncture access kit (dashed arrow); (B) Enroute™ 8Fr sheath placed through lower neck cutdown in the common carotid artery (CAA). Note the CCA is clamped while flow reversal is ensured; (C) Left internal carotid artery (ICA) focal, near total occlusion (black arrow) at the level of C1; (D) Completion angiogram showing resolution of ICA stenosis.

The most recent neuroprotection device approved by the FDA is the ENROUTE™ system (Silk Road Medical, Sunnyvale, California). The common carotid artery (CCA) is dissected out and controlled with vessel loops followed by insertion of a special sheath in the CCA; the CCA is then clamped and flow reversal started through the ENROUTE™ system. (Figure 1). Its safety and efficacy was first reported in a single-center German study, the PROOF study, which enrolled 75 patients; the majority of these patients were asymptomatic (63, 84%).^16^ Five (7%) patients had transient intolerance to flow reversal, which did not hinder completion of the procedure. Outstanding results of no death, no major strokes, no MI, and only one minor stroke, which was not related to the device or procedure were reported. A multicenter, prospective trial, the Safety and Efficacy Study for Reverse Flow Used During Carotid Artery Stenting Procedure (ROADSTER) to evaluate the safety and efficacy of ENROUTE Transcarotid NPS (Silk Road Medical, Sunnyvale, CA), was recently published.^14^ Similar to the PROOF study, the 141 patients’ outcomes were outstanding and unprecedented with 1.4% (2 of 141) all-stroke rate, 2.8% (4 of 141) stroke and death rate, and 3.5% (5 of 141) stroke, death, and MI rate. By far, these numbers are the lowest reported to date for any prospective, multicenter clinical trial of CAS.

Transcarotid artery stenting has gained traction over the past two decades; its contemporary data has shown stroke rates as low as 1.4%. The major drawbacks of this technique are the lack of randomized controlled trials, limitations of its indications (i.e. carotid artery dissection), and cost in countries with limited healthcare funding. Despite these shortcomings, TCAR will become more accessible and popular in the years to come as a safe and efficacious alternative to carotid artery endarterectomy and mainly to transfemoral carotid artery stenting to treat both asymptomatic and symptomatic carotid artery disease.
